# Supplementary material for: ChatGPT as a Tool for Biostatisticians: A Tutorial on Applications, Opportunities, and Limitations
Source: Stat Med. 2025 Oct 23;44(23-24):e70263. doi: 10.1002/sim.70263 (PMC12548020; doi:10.1002/sim.70263)
Supplement: Supplementary file 1 — Data S1. Supporting Information. [file SIM-44-0-s001.zip › Supplementary Materials Additional.zip!/3.7_Translation_Between_Multiple_Statistical_Programming_Languages/3.7.1_R_to_python/3.7.1_Translation_Chat3.pdf]

Run 3

Clear memory

I don't have memory on in this chat, so I can't remember anything we talk about across sessions.

If you'd like to clear memory from past interactions (when memory *is* on), you can do that by going to **Settings > Personalization > Memory** and managing or clearing memory there.

Want help getting to that setting?

Take the role of an expert biostatistician for this chat

Got it — stepping into expert biostatistician mode. Whether you're diving into survival analysis, GLMs, mixed models, clinical trial design, or wrangling messy data, I've got you covered.

What are we tackling today?

Translate the following code into python:

```
library(MASS)
set.seed(1)
temp <- tempfile()
download.file(
  "https://archive.ics.uci.edu/static/public/17/breast+cancer+wisconsin+diagnostic.zip",
  temp
)
data <- read.table(unz(temp, "wdbc.data"), sep = ",")
data$V2 <- data$V2 == "M"
unlink(temp)

train_index <- sample(seq_len(nrow(data)), round(nrow(data) * 0.8))

train <- data[train_index, -1]
test <- data[-train_index, -1]

base_model <- glm(V2 ~ 1, data = train, family = binomial)


step_model <- stepAIC(
  base_model,
  direction = "both",
```

```
  scope = list(
    lower = V2 ~ 1,
    upper = V2 ~ V3 + V4 + V5 + V6 + V7 + V8 + V9 + V10 + V11 +
      V12 + V13 + V14 + V15 + V16 + V17 + V18 + V19 + V20 + V21 +
      V22 + V23 + V24 + V25 + V26 + V27 + V28 + V29 + V30 + V31
  ),
  # scope = formula(V2 ~ .),
)

summary(step_model)
test_pred <- predict(step_model, test, type = "response")

sum((test_pred > 0.5) == test$V2) / nrow (test)
names(coef(step_model))
```

Here’s a faithful Python translation of your R code using standard packages like **`pandas`**, **`statsmodels`**, and **`sklearn`**. R's **`stepAIC()`** for stepwise selection doesn’t have a direct equivalent in base Python, so we’ll use **`statsmodels`** and **`mlxtend`** to approximate that behavior.

```python
import pandas as pd
import numpy as np
import requests, zipfile, io
from sklearn.model_selection import train_test_split
import statsmodels.api as sm
from mlxtend.feature_selection import SequentialFeatureSelector as SFS
from sklearn.linear_model import LogisticRegression
from sklearn.metrics import accuracy_score

# Set seed for reproducibility
np.random.seed(1)

# Download and load the dataset
url = "https://archive.ics.uci.edu/static/public/17/breast+cancer+wisconsin+diagnostic.zip"
r = requests.get(url)
z = zipfile.ZipFile(io.BytesIO(r.content))
df = pd.read_csv(z.open("wdbc.data"), header=None)

# Convert target variable to binary
df[1] = df[1] == "M"

# Drop the ID column and split into train/test sets
X = df.drop(columns=[0, 1])
y = df[1]
X_train, X_test, y_train, y_test = train_test_split(X, y, test_size=0.2, random_state=1)

# Fit base logistic regression model (intercept only)
X_train_const = sm.add_constant(pd.DataFrame(np.ones((X_train.shape[0], 1)), columns=["const"]))
base_model = sm.Logit(y_train, X_train_const).fit(disp=0)

# Stepwise selection using mlxtend
lr = LogisticRegression(solver='liblinear')
sfs = SFS(lr,
          k_features='best',
          forward=True,
          floating=True,
          scoring='accuracy',
          cv=5,
          n_jobs=-1)

sfs = sfs.fit(X_train, y_train)

# Fit model with selected features
```

```
selected_features = list(sfs.k_feature_names_)
X_train_selected = X_train[selected_features]
X_test_selected = X_test[selected_features]

final_model = sm.Logit(y_train, sm.add_constant(X_train_selected)).fit(disp=0)

# Predict on test data
test_pred_prob = final_model.predict(sm.add_constant(X_test_selected))
test_pred_class = test_pred_prob > 0.5

# Accuracy
accuracy = accuracy_score(y_test, test_pred_class)
print("Accuracy:", accuracy)

# Coefficients
print("Selected features and coefficients:")
print(final_model.params)
```

Notes:

- `` `mlxtend` ``'s SFS is used here to mimic stepwise selection.
- `` `statsmodels.Logit` `` gives interpretable coefficients, like R's `` `glm()` ``.
- You could switch to `` `sklearn.linear_model.LogisticRegression` `` throughout if you don't need p-values or summary stats.

Would you like a version that exactly mimics R's bidirectional stepwise AIC (including backward elimination)?

The line
base_model = sm.Logit(y_train, X_train_const).fit(disp=0)
throws error
ValueError: The indices for endog and exog are not aligned

Great catch — that error happens because the indices of `` `y_train` `` and `` `X_train_const` `` don't match. In `` `statsmodels` ``, both `` `endog` `` (i.e., `` `y_train` ``) and `` `exog` `` (i.e., predictors) must have aligned indices.

You can fix this by resetting the index on both objects before fitting the model. Here's the corrected version of that line:

```python
# Reset indices to align exog and endog
y_train_reset = y_train.reset_index(drop=True)
X_train_const = pd.DataFrame({'const': 1}, index=y_train_reset.index)

# Fit base model
base_model = sm.Logit(y_train_reset, X_train_const).fit(disp=0)
```

That ensures the constant-only design matrix (`` `X_train_const` ``) and the target variable (`` `y_train` ``) are properly aligned.

Let me know if you also want the **stepwise AIC-style selection** implemented manually in Python — it's a bit more involved, but totally doable.

Implement the stepwise AIC-style selection instead

Perfect — let's implement **stepwise selection using AIC** manually in Python, just like `stepAIC()` in R. This will include:

- A base (intercept-only) model.
- A loop that adds or removes variables one at a time.
- Forward and backward steps.
- Using **AIC** from `statsmodels.Logit` for decision making.

Here's a full implementation using your dataset:

```python
import pandas as pd
import numpy as np
import requests, zipfile, io
import statsmodels.api as sm
from sklearn.model_selection import train_test_split
from sklearn.metrics import accuracy_score

# Set seed
np.random.seed(1)

# Download and load the dataset
url = "https://archive.ics.uci.edu/static/public/17/breast+cancer+wisconsin+diagnostic.zip"
r = requests.get(url)
z = zipfile.ZipFile(io.BytesIO(r.content))
df = pd.read_csv(z.open("wdbc.data"), header=None)

# Convert target variable to binary
df[1] = df[1] == "M"

# Drop ID column and split data
X = df.drop(columns=[0, 1])
y = df[1]
X_train, X_test, y_train, y_test = train_test_split(X, y, test_size=0.2, random_state=1)

# Reset indices
X_train = X_train.reset_index(drop=True)
y_train = y_train.reset_index(drop=True)
X_test = X_test.reset_index(drop=True)
y_test = y_test.reset_index(drop=True)

# Stepwise AIC
def calculate_aic(model):
    return model.aic

def fit_model(features):
    X_with_const = sm.add_constant(X_train[features]) if features else pd.DataFrame({'const': 1}, i
    model = sm.Logit(y_train, X_with_const).fit(disp=0)
    return model

def stepwise_selection():
    remaining = set(X_train.columns)
    selected = []
    current_score, best_new_score = np.inf, np.inf
    while True:
        scores_with_candidates = []

        # Forward step
        for candidate in remaining:
```

```
            model = fit_model(selected + [candidate])
            aic = calculate_aic(model)
            scores_with_candidates.append((aic, candidate))

        scores_with_candidates.sort()
        best_new_score, best_candidate = scores_with_candidates[0]

        if best_new_score < current_score:
            remaining.remove(best_candidate)
            selected.append(best_candidate)
            current_score = best_new_score
        else:
            break

        # Backward step
        while len(selected) > 1:
            scores_with_removals = []
            for candidate in selected:
                temp_features = [f for f in selected if f != candidate]
                model = fit_model(temp_features)
                aic = calculate_aic(model)
                scores_with_removals.append((aic, candidate))

            scores_with_removals.sort()
            best_removal_score, worst_feature = scores_with_removals[0]

            if best_removal_score < current_score:
                selected.remove(worst_feature)
                current_score = best_removal_score
            else:
                break

    return selected

# Perform stepwise selection
selected_features = stepwise_selection()
print("Selected features:", selected_features)

# Fit final model
final_model = fit_model(selected_features)
print(final_model.summary())

# Predict and evaluate
X_test_selected = sm.add_constant(X_test[selected_features])
test_pred_prob = final_model.predict(X_test_selected)
test_pred_class = test_pred_prob > 0.5

accuracy = accuracy_score(y_test, test_pred_class)
print("Test accuracy:", accuracy)

# List coefficients
print("Model coefficients:")
print(final_model.params)
```

Key Notes:

- This version mimics both **forward selection** and **backward elimination** based on **AIC**.
- It starts from the intercept-only model and evaluates AIC when adding or removing features.
- The loop stops when no further AIC improvement is observed.

Would you like to add regularization (like LASSO) or cross-validation for model robustness as a next step?
